# Temporal course of cognitive and behavioural changes in motor neuron diseases

**DOI:** 10.1136/jnnp-2023-331697

**Published:** 2023-10-12

**Authors:** Caroline A McHutchison, Joanne Wuu, Corey T McMillan, Rosa Rademakers, Jeffrey Statland, Gang Wu, Evadnie Rampersaud, Jason Myers, Jessica P Hernandez, Sharon Abrahams, Michael Benatar

**Affiliations:** 1 School of Philosophy, Psychology, and Language Sciences, The University of Edinburgh, Edinburgh, UK; 2 Euan MacDonald Centre for Motor Neuron Disease Research, The University of Edinburgh, Edinburgh, UK; 3 Department of Neurology, University of Miami Miller School of Medicine, Miami, Florida, USA; 4 Department of Neurology, University of Pennsylvania Perelman School of Medicine, Philadelphia, Pennsylvania, USA; 5 Department of Biomedical Sciences, University of Antwerp, Antwerp, Belgium; 6 Department of Neurology, The University of Kansas Medical Center, Kansas City, Kansas, USA; 7 Department of Computational Biology, St Jude Children's Research Hospital, Memphis, Tennessee, USA

**Keywords:** COGNITION, ALS, C9ORF, MOTOR NEURON DISEASE, FRONTOTEMPORAL DEMENTIA

## Abstract

**Background:**

Cognitive and behavioural dysfunction may occur in people with motor neuron disease (MND), with some studies suggesting an association with the *C9ORF72* repeat expansion. Their onset and progression, however, is poorly understood. We explored how cognition and behaviour change over time, and whether demographic, clinical and genetic factors impact these changes.

**Methods:**

Participants with MND were recruited through the Phenotype-Genotype-Biomarker study. Every 3–6 months, the Edinburgh Cognitive and Behavioural ALS Screen (ECAS) was used to assess amyotrophic lateral sclerosis (ALS) specific (executive functioning, verbal fluency, language) and ALS non-specific (memory, visuospatial) functions. Informants reported on behaviour symptoms via semi-structured interview.

**Results:**

Participants with neuropsychological data at ≥3 visits were included (n=237, mean age=59, 60% male), of which 18 (8%) were *C9ORF72* positive. Baseline cognitive impairment was apparent in 18 (8%), typically in ALS specific domains, and associated with lower education, but not *C9ORF72* status. Cognition, on average, remained stable over time, with two exceptions: (1) *C9ORF72* carriers declined in all ECAS domains, (2) 8%–9% of participants with baseline cognitive impairment further declined, primarily in the ALS non-specific domain, which was associated with less education. Behavioural symptoms were uncommon.

**Conclusions:**

In this study, cognitive dysfunction was less common than previously reported and remained stable over time for most. However, cognition declines longitudinally in a small subset, which is not entirely related to *C9ORF72* status. Our findings raise questions about the timing of cognitive impairment in MND, and whether it arises during early clinically manifest disease or even prior to motor manifestations.

**WHAT IS ALREADY KNOWN ON THIS TOPIC**

- The onset and progression of cognitive and behavioural symptoms in motor neuron diseases is poorly understood, with previous studies showing mixed results.

**WHAT THIS STUDY ADDS**

- In our large observational study, we showed that cognitive impairment at initial assessment was infrequent, most often involves domains typically affected in amyotrophic lateral sclerosis (executive functioning, language, verbal fluency), and remains stable over time for most patients. However, a small group show decline on all cognitive domains, and this is not entirely explained by the *C9ORF72* repeat expansion.

**HOW THIS STUDY MIGHT AFFECT RESEARCH, PRACTICE OR POLICY**

- This highlights the need for future research to identify when these cognitive symptoms begin, relative to motor symptom onset, and what other factors are associated with decline over time.

## Introduction

Cognitive and behavioural symptoms have been reported to occur in many patients with amyotrophic lateral sclerosis (ALS), the most common form of motor neuron disease (MND), with frontotemporal dementia, typically the behavioural variant (bvFTD) occurring in ~5%–15% of people with ALS. This has prompted proposal of the term: ALS frontotemporal spectrum disorder (ALS-FTSD).^1^ Deficits in executive functioning, verbal fluency and language are the typical cognitive symptoms, but memory deficits may also be evident. While apathy is the most common behavioural symptom,^2 3^ other FTSD-related symptoms may include disinhibition, loss of sympathy and/or empathy, perseveration and hyperorality. Neuropsychological symptoms in ALS are associated with poor quality of life, worse prognosis, increased caregiver burden,^4–6^ and may impact patients’ capacity to make decisions regarding their care and treatment adherence may be compromised.^7^

Although the nature of cognitive and behavioural deficits in ALS is well described, our understanding of the onset and progression of these symptoms, relative to motor manifestations of disease, remains unclear. Cross-sectional studies have suggested that cognitive impairment increases with advancing disease stage,^8 9^ particularly in functions more typically affected in ALS (language, executive functioning and verbal fluency).^8^ Of 13 longitudinal studies, 62% showed stable cognition over periods of ~6–24 months, while 31% suggested that certain patients show decline,^10^ a pattern associated with executive dysfunction^4^ and shorter disease duration at initial evaluation.^11 12^ However, much of the research to date has been hampered by small sample sizes, high attrition rates and limited follow-up assessments. Neuropsychological assessment has also previously been limited by the lack of tests suitable for those with motor symptoms and repeated administration. Furthermore, the updated ALS-FTSD criteria^1^ allows for more accurate and reliable delineation of cognitive and/behavioural impairment in ALS.

Identification of the genetic contribution to ALS and FTD, most notably the *C9ORF72* repeat expansion, has buttressed the clinical and pathological evidence for the overlap between ALS and FTD. The *C9ORF72* mutation has been reported in approximately 6.3% and 5.8% of sporadic ALS and FTD cases, respectively,^13^ and 33% of familial ALS cases.^14^ An association between the presence of *C9ORF72* and cognitive impairment has been reported in some, but not all, ALS studies.^15 16^ It is unclear whether these differences may relate to varying prevalence of genetic modifiers such as *TMEM106B* and *UNC13A*. Evidence for protection against cognitive impairment in the presence of protective alleles at these genetic loci has previously been suggested in both ALS^17^ and FTD.^18 19^

Here we investigated whether cognitive and behavioural symptoms in MND change over time and if so, what factors (demographic, clinical and genetic) are associated with these changes. We also examined whether different subgroups were present representing different patterns of cognitive change.

## Materials and methods

### Study population

Patients diagnosed with ALS (with or without FTD), primary lateral sclerosis (PLS), progressive muscular atrophy (PMA), hereditary spastic paraplegia or multisystem proteinopathy were enrolled in the multi-centre longitudinal Phenotype-Genotype-Biomarker (PGB) study of the Clinical Research in ALS and Related Disorders for Therapeutic Development (CReATe) consortium between 2014 and 2019. Following baseline assessment, participants were evaluated every 3–6 months for up to five visits. Rigorously standardised clinical assessments and biological sample collections were performed at each visit.

### Genetic analysis

All participants underwent analysis of *C9ORF72* repeat expansion via a combination of a fluorescent assay and repeat-primed PCR assay, as well as whole genome sequencing (WGS) with curation for known pathogenic variants and Sanger validation as needed. Single nucleotide polymorphisms (SNPs) in relevant genes such as transmembrane protein 106B (*TMEM106B*: rs3173615 and rs1990622) and *UNC13A* (rs12608932) were extracted from WGS data. *TMEM106B* and *UNC13A* genotypes not meeting quality threshold (>20× depth) were removed. Due to high linkage disequilibrium between rs3173615 and rs1990622, only rs3173615 and rs12608932 were examined in analysis.

### Neuropsychological evaluations

Cognition and behaviour were assessed at each visit using one of the three alternate versions (Form A, B and C) of the Edinburgh Cognitive and Behavioural ALS Screen (ECAS).^20 21^ The ECAS is a multi-domain cognitive assessment developed specifically for use in the ALS population. It assesses ALS specific functions typically affected (language, verbal fluency and executive functions) and ALS non-specific functions, less likely to be affected in ALS but commonly affected in other disorders of older adults (memory and visuospatial functions); it has been shown to have high sensitivity and specificity against full neuropsychological assessment^22^; and published reliable change indices aid interpretation of repeated assessments.^23^ A behaviour screen assesses five domains of behavioural change based on the Rascovsky criteria for the diagnosis of bvFTD (behavioural disinhibition, apathy, loss of sympathy/empathy, perseverative behaviour and hyperorality/changes in food preferences)^24^ and is administered as a semi-structured interview with an informant. All interviews were conducted by a trained research assistant either in-person or over the phone. Additional details provided by the informant were recorded and reviewed by members of the study team to ensure that behaviours of interest were captured. Moreover, the ECAS has been adapted for several languages which were used in the CReATe PGB study, with most participants being English-speakers who completed the North American English ECAS.

Participants were classified at each visit as having cognitive impairment (ALSci), behavioural impairment (ALSbi) or both (ALScbi) according to the ALS-FTSD criteria.^1^ PLS and PMA participants were categorised using the same criteria based on evidence that these subtypes have neuropsychological profiles similar to ALS.^25^ Cognitive impairment was determined using the ECAS North American quantile regression norms.^26^

### Statistical analysis

#### Inclusion/exclusion

The analysis dataset included North American English ECAS data collected from participants with a diagnosis of ALS, PMA or PLS, henceforth encompassed by the term MND. Those with a diagnosis of FTD (or ALS-FTD) at time of first ECAS assessment were excluded (n=5). Participants with ECAS data at three or more time points comprised the longitudinal cohort.

#### Baseline analysis

Baseline cognition (based on first ECAS assessment), demographic and clinical factors were compared between those in the longitudinal cohort versus those who completed fewer than three ECAS assessments, using χ^2^ (categorical) and t-test or Mann-Whitney U test (continuous). Baseline ALS Functional Rating Scale-Revised (ALSFRS-R) scores were used to estimate the rate of decline in functional ability prior to baseline (ΔFRS: 48–baseline ALSFRS-R/months from onset to baseline).^27^ The association between demographic/clinical factors and baseline ECAS cognitive performance was examined using multiple linear regression.

#### Longitudinal analysis

Analysis of longitudinal cognitive data was conducted using linear mixed effects models, with time defined as months from baseline. Models included by-participant random intercepts and slopes of time, fitted with an unstructured covariance matrix. Three sets of analyses were performed, with ECAS ALS specific, ALS non-specific or total scores as the dependent variable. To explore potential baseline predictors of subsequent cognitive change, we considered demographic characteristics (age, sex, education) and clinical/genetic factors (baseline cognitive impairment, bulbar onset, estimated progression rate from symptom onset to baseline (∆FRS), and *C9ORF72* status). Interactions between each of these variables and time were examined, and only those where likelihood ratio tests (derived using parametric bootstrapping) indicated that they improved model fit were included in the final model (*C9ORF72* status for all ECAS outcomes with the addition of education for ALS specific scores). The rate of cognitive change was further characterised separately for *C9ORF72* expansion carriers and non-carriers using linear mixed effect models. The effect of potential genetic modifiers (in *TMEM106B* or *UNC13A*) on cognitive trajectories for *C9ORF72* expansion carriers and non-carriers were examined under minor allele recessive and additive models.

The presence of subgroups representing different patterns of cognitive change was explored using latent class growth analysis (LCGA). For each ECAS measure, models with one to four subgroups were compared using indices of model fit (Bayesian information criterion and Akaike information criterion), class separation (degree to which classes overlap) and visualisation of modelled trajectories. Predictors (demographic, clinical and genetic factors) of class membership were included using a one-step approach. Comparison of mixing probabilities for models with and without predictors suggested minimal model distortion, supporting stability of the models. We used the class with the highest baseline scores as the reference group as it represents cognitive functioning within the normal range.

All analyses were conducted in R^28^ and models were fitted using the lme4 package^29^ with the bobyqa optimiser (mixed effects) and the lccm package (LCGA).^30^ Mixed effects models employed the Satterthwaite approximation to df. Where appropriate, adjustments for multiple testing were conducted using the Holm method.

## Results

North American English ECAS data were available for n=423 participants (total cohort), of whom n=237 (56%) had ECAS data at three or more time points (longitudinal cohort; tables 1 and 2). Compared with the longitudinal cohort, participants with ECAS data at one or two time points only (≤2 time points cohort; n=186) had significantly lower ALSFRS-R scores, faster rates of disease progression before baseline (ΔFRS of 0.49 vs 0.33, p<0.001) and were more likely to have cognitive impairment (ALSci or ALScbi) at initial assessment (n=33 (18%) vs n=18 (8%); p=0.002; see online supplemental eTables 1 and 2 for more details), with worse average scores on ALS specific, ALS non-specific and ECAS total scores. There were no significant differences on any other demographic or clinical characteristics. Disease progression was the most common reason for having fewer than three ECAS assessments (n=82), followed by lost to follow-up (n=50), and early study closure due to administrative reason (n=49). A further five participants continued study visits but four did not complete ECAS assessment (participant fatigue (n=2), remote visit (n=2)), and the assessment was invalid for one participant.

10.1136/jnnp-2023-331697.supp1 Supplementary data

**Table 1:** Baseline characteristics of longitudinal cohort^*^

	ALS	PLS	PMA	All diagnoses
	(n=207)	(n=21)	(n=9)	(n=237)
Age (years)	59.1±11.2	61.6±10.7	52.3±11.4	59.1±11.2
Sex, male	125 (60%)	12 (57%)	6 (67%)	143 (60%)
Education (years)	15.7±3.2	15.9±3.2	15.1±2.0	15.6±3.1
*C9ORF72* expansion carrier	18 (9%)	0 (0%)	0 (0%)	18 (8%)
Bulbar symptoms at onset	50 (24%)	6 (29%)	0 (0%)	56 (24%)
Symptom onset to baseline (months)	27.3 (14.3, 49.7)	103.5 (56.2, 145.3)	123.8 (54.6, 126.9)	31.5 (15.8, 63.7)
Baseline ALSFRS-R	37 (32, 41)	35 (32, 38)	36 (33, 42)	37 (32, 41)
Baseline ΔFRS (points/month)^†^	0.34 (0.21, 0.63)	0.13 (0.08, 0.25)	0.12 (0.07, 0.22)	0.33 (0.17, 0.59)

Values are mean±SD; median (25th percentile, 75th percentile); or n (%).

*Clinical Research in ALS and Related Disorders for Therapeutic Development Phenotype-Genotype-Biomarker study participants who completed the North American English version of the Edinburgh Cognitive and Behavioural ALS Screen at three or more time points.

†ΔFRS=(48–baseline ALSFRS-R)/months from symptom onset to baseline.

ALS, amyotrophic lateral sclerosis; ALSFRS-R, ALS Functional Rating Scale-Revised; FRS, Functional Rating Scale.

**Table 2:** Baseline ECAS scores of longitudinal cohort^*^

ECAS: cognition	Max	ALS	PLS	PMA	All diagnoses
		(n=207)	(n=21)	(n=9)	(n=237)
Language	28	26.1±2.5	26.9±1.0	26.4±1.5	26.2±2.4
Verbal fluency	24	17.5±4.6	19.5±2.9	17.8±2.7	17.7±4.5
Executive functioning	48	39.8±5.0	40.0±5.8	38.8±5.1	39.8±5.1
*ALS specific score*	*100*	*83.4±8.8*	*86.4±7.5*	*83.0±6.9*	*83.6±8.7*
Memory	24	16.8±3.1	17.3±2.7	15.7±6.7	16.8±3.3
Visuospatial	12	11.6±0.9	11.6±0.7	11.9±0.3	11.6±0.9
*ALS non-specific score*	*26*	*28.4±3.4*	*28.9±2.9*	*27.6±6.7*	*28.4±3.5*
*ECAS total score*	*136*	*111.8±10.4*	*115.3±9.8*	*110.6±12.2*	*112.0±10.5*

ECAS: behaviour^†^		(n=134)	(n=13)	(n=5)	(n=152)
Disinhibition	–	8 (6%)	0 (0%)	0 (0%)	8 (5%)
Apathy	–	21 (16%)	0 (0%)	0 (0%)	21 (14%)
Loss of sympathy/empathy	–	16 (12%)	2 (15%)	0 (0%)	18 (12%)
Perseveration	–	6 (4%)	1 (8%)	0 (0%)	7 (5%)
Hyperorality	–	7 (5%)	0 (0%)	0 (0%)	7 (5%)
ALS-FTSD classification: North American quantile regression cut-offs^‡^					
ALSci		13 (6%)	1 (5%)	1 (11%)	15 (6%)
ALSbi		22 (11%)	1 (5%)	0 (0%)	23 (10%)
ALScbi		3 (1%)	0 (0%)	0 (0%)	3 (1%)
ALS-FTSD classification: UK 2 SD cut-offs^‡^					
ALSci		47 (23%)	4 (19%)	3 (33%)	54 (23%)
ALSbi		16 (8%)	1 (5%)	0 (0%)	17 (7%)
ALScbi		9 (4%)	0 (0%)	0 (0%)	9 (4%)

Values are mean±SD; median (25th percentile, 75th percentile); or n (%).

*CReATe PGB study participants who completed the North American English version of the ECAS at three or more time points.

†n=85 did not have ECAS behaviour data available at baseline.

‡Classified based on available ECAS data for each participant. PLS and PMA participants were also classified using the same criteria based on prior evidence of comparable neuropsychological profiles. Participants were not classified as ALS-FTD based on ECAS scores as this requires clinical observations/judgements of change over time.^1^

ALSbi, amyotrophic lateral sclerosis with behavioural impairment; ALScbi, amyotrophic lateral sclerosis with cognitive and behavioural impairment; ALSci, amyotrophic lateral sclerosis with cognitive impairment; ALS-FTD, amyotrophic lateral sclerosis with frontotemporal dementia; CReATe, Clinical Research in ALS and Related Disorders for Therapeutic Development; ECAS, Edinburgh Cognitive and Behavioural ALS Screen; PGB, Phenotype-Genotype-Biomarker; PLS, primary lateral sclerosis; PMA, progressive muscular atrophy.

### Baseline cognition

Of the 237 participants in the longitudinal cohort, 6% (n=15) were classified as ALSci, 10% (n=23) as ALSbi and 1% (n=3) as ALScbi at baseline (table 2). Of the n=18 with any cognitive impairment (ALSci or ALScbi) at first assessment, 1 (8%) carried the *C9ORF72* repeat expansion, 83% (n=15) were impaired on the ALS specific domain (10 on the ALS specific domain only, 5 on both ALS specific and non-specific domains) and 17% (n=3) on the ALS non-specific domain only. Adjusting for baseline age, sex, bulbar onset and ΔFRS in the model, lower ECAS scores at baseline were associated with older age and lower education: for every 10 additional years in age, ALS specific and non-specific scores were on average lower by 1.3 and 0.6 points, respectively; and for every four additional years of education, ALS specific and non-specific scores were higher by 2.2 and 0.7 points, respectively (table 3). Similar results were found for ECAS total scores.

**Table 3:** Baseline and longitudinal changes in ECAS

ALS specific		ALS non-specific		ECAS total	
**Factors associated with cognition at first assessment**					
Age (years)	ß=−0.13 (0.05)^*^ 95% CI −0.23 to –0.04	Age (years)	ß=−0.06 (0.02)^*^ 95% CI −0.10 to –0.02	Age (years)	ß=−0.19 (0.06)^*^ 95% CI −0.31 to –0.07
Education (years)^‡^	ß=2.15 (0.71)^*^ 95% CI 0.79 to 3.57	Education (years)^‡^	ß=0.70 (0.28)^*^ 95% CI 0.20 to 1.34	Education (years)^‡^	ß=2.85 (0.87)^*§^ 95% CI −1.26 to 4.71
**Changes in cognition over time for all participants**					
Did not significantly change	ß=−0.03 (0.03) 95% CI −0.09 to 0.03	Increased	ß=0.04 (0.02)^*^ 95% CI 0.01 to 0.07	Did not significantly change	ß=0.01 (0.04) 95% CI −0.07 to 0.08
**Changes in cognition over time based on** ***C9ORF72*** **status** ^**¶**^					
C9ORF72 positive	ß=−0.53 (0.29) 95% CI −1.10 to 0.04	C9ORF72 positive	ß=−0.14 (0.06)^*^ 95% CI −0.27 to –0.02	C9ORF72 positive	ß=−0.65 (0.34) 95% CI −1.35 to 0.05
C9ORF72 negative	ß=−0.01 (0.03) 95% CI −0.07 to 0.05	C9ORF72 negative	ß=0.05 (0.01)^†^ 95% CI 0.02 to 0.08	C9ORF72 negative	ß=0.04 (0.04) 95% CI −0.04 to 0.12
**Factors associated with low baseline-declining pattern of cognitive change** ^**^					
Education (years) ^‡^	OR=0.31 95% CI 0.14, 0.73 ^*^			Education (years) ^‡^	OR=0.39 95% CI 0.16, 0.93 ^*^

Values are beta coefficients (SE) and 95% CI or odd ratios (OR) and 95% CI.

All models were adjusted for baseline ∆FRS, baseline age, sex, education, bulbar symptoms at onset and *C9ORF72* status unless otherwise specified.

*p<0.05.

†p<0.001.

‡In 4-year increments.

§Significant after correction for multiple testing using the Holm method.

¶Adjusted for baseline age and ∆FRS.

**Relative to belonging to the high baseline-upward group.

ALS, amyotrophic lateral sclerosis; ECAS, Edinburgh Cognitive and Behavioural ALS Screen; FRS, Functional Rating Scale.

### Cognitive changes over time

**Figure 1:**
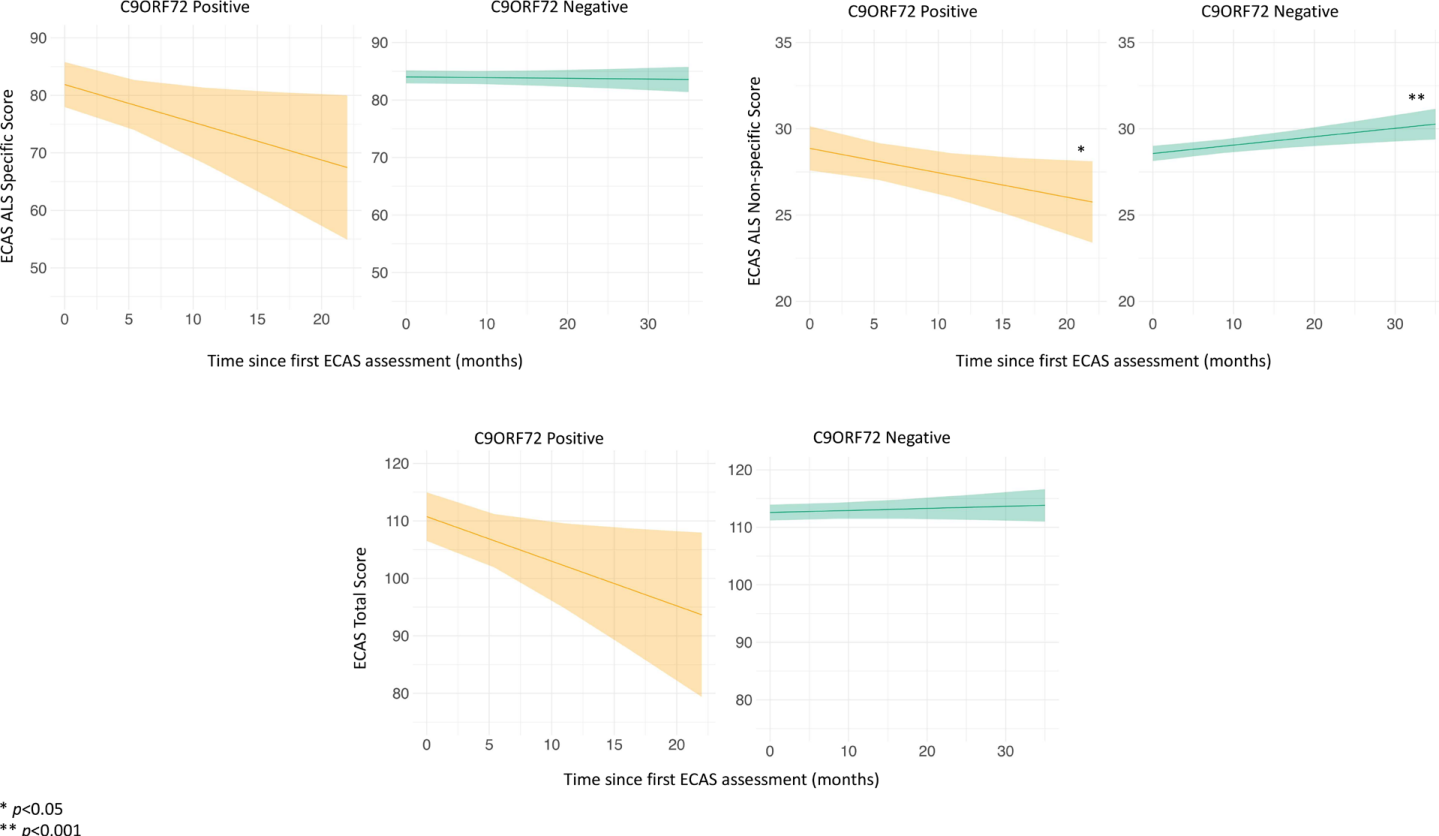
Patterns of cognitive change stratified by *C9ORF72* status for each ECAS measure. ALS, amyotrophic lateral sclerosis; ECAS, Edinburgh Cognitive and Behavioural ALS Screen.

In the longitudinal cohort (n=237), the increase in ALS non-specific scores was, on average, small but statistically significant (0.04 points/month, p=0.008). ALS specific scores and total scores did not change over time (table 3). However, cognitive trajectories differed between those with and without a *C9ORF72* repeat expansion. Among *C9ORF72* expansion carriers cognitive function declined on all three ECAS scores, but with the rate of decline in ALS specific scores mitigated by higher levels of education (online supplemental eTable 3). Specifically, *C9ORF72* expansion carriers declined, on average, by 0.53 (ALS specific), 0.14 (ALS non-specific) and 0.65 (ECAS total) points/month. By contrast, cognitive function remained stable or even slightly improved among those without a *C9ORF72* repeat expansion (figure 1, table 3). We found no evidence that baseline cognitive impairment was a predictor of subsequent cognitive decline or that cognitive trajectories differed based on the presence of *TMEM106B* rs3173615 or *UNC13A* rs12608932 genetic modifiers, among *C9ORF72* expansion carriers or non-carriers (see online supplemental eTable 4 for SNP frequencies, other data not shown).

**Figure 2:**
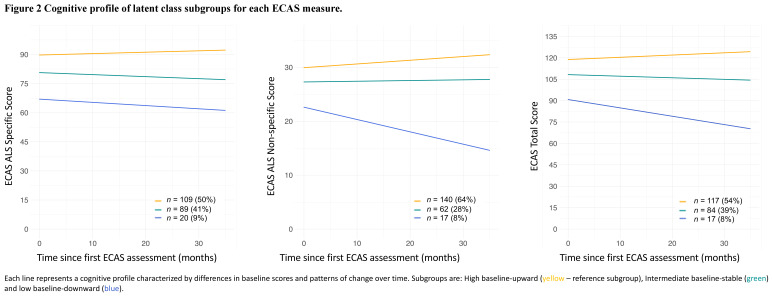
Subgroup specific patterns of cognitive change for each ECAS measure. ALS, amyotrophic lateral sclerosis; ECAS, Edinburgh Cognitive and Behavioural ALS Screen.

For each ECAS measure, LCGA identified three distinct subgroups of individuals representing different patterns of cognitive change over time (figure 2 and online supplemental eTable 5). The longitudinal cognitive profile of a small proportion (8%–9%) of study participants was characterised by cognitive functioning that was, on average, impaired at first assessment and significantly declined over time (low baseline-downward subgroup, shown in blue in figure 2). These longitudinal changes were observed for all three ECAS measures but were most marked for the ALS non-specific (−0.2 points/month) and ECAS total (−0.6 points/month) scores. Lower education was associated with this cognitive profile for ALS specific and ECAS total scores, but not ALS non-specific (relative to high baseline-upward; table 3 and online supplemental eTable 5) but was not associated with *C9ORF72* status.

### Behaviour

**Figure 3:**
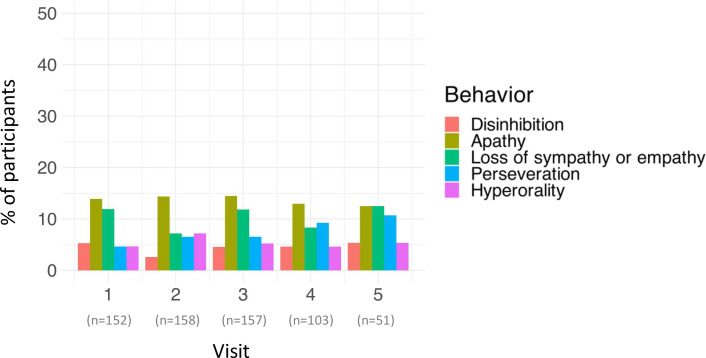
Proportion of participants at each visit with each behaviour symptom.

**Figure 4:**
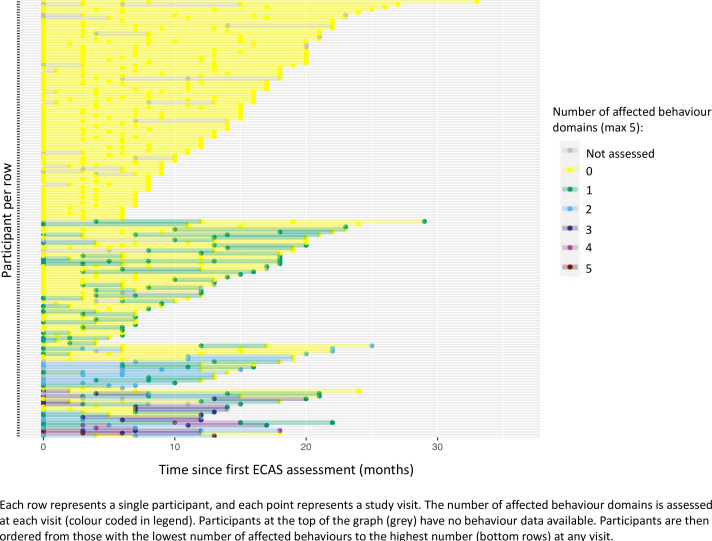
Changes in the number of affected behaviour domains over time. ALS, amyotrophic lateral sclerosis; ECAS, Edinburgh Cognitive and Behavioural ALS Screen.

From the full dataset (n=423), 39% (n=165) had ECAS behaviour data at three or more time points (all of which were included in the cognitive longitudinal cohort), with behavioural impairment (ALSbi or ALScbi) at initial assessment present in 16% (n=26). Apathy was the most prevalent behavioural symptom at all time points, and disinhibition was the least (figure 3). The number of reported behavioural symptoms (maximal 5) fluctuated over time, but rates were generally low (figure 4). At any point in time over follow-up, most participants had no affected behavioural domains (n=83) or a maximum of one affected domain (n*=*47). A small number of participants had two (n=17), with the number of affected domains remaining mostly stable, while very few (n=12) had three domains affected. The presence of four or five affected behaviour domains at any study visit was uncommon (both n=3). At the final visits, four (2%) participants were clinically diagnosed with ALS-FTD by a neurologist. Persistent behavioural symptoms, defined as a symptom being present at two or more consecutive timepoints, were most common for apathy (16 (10%)) and loss of sympathy and/or empathy (11 (7%)). Persistent perseveration (7 (4%)), hyperorality (7 (4%)) and disinhibition (5 (3%))) were least common.

## Discussion

The CReATe Consortium’s PGB study represents one of the largest observational studies with longitudinal cognitive and behavioural data collected using an instrument suitable for use in the ALS population. Our findings suggest that when cognitive impairment is present at the initial assessment, it most often involves language and executive functioning (ALS specific domains) and is associated with lower educational levels, but not with the *C9ORF72* repeat expansion. Cognitive function, on average, does not decline in a clinically meaningful way over time, with two exceptions. First, among those with a *C9ORF72* repeat expansion, there is cognitive decline in both ALS-specific and ALS non-specific domains over time. Second, a small group of patients (8%–9%) with lower education levels, show impaired cognition at baseline and continued decline over time across all measures, but most notably ALS non-specific domains. Importantly, and in contrast to some of the published literature,^17 18 31^ there is no clear beneficial effect of the putative protective alleles in *UNC13A* and *TMEM106B* on cognitive decline, either among those with or without the *C9ORF72* repeat expansion. Finally, at least one behavioural symptom at any point was evident in around 50% of participants, however few met criteria for behavioural impairment with fluctuations in behavioural function over time.

In contrast to previous research, cognitive impairment was relatively uncommon in our study population (8%–18%) and remained infrequent at all visits. Importantly we determined cognitive impairment based on the more conservative North American ECAS cut-offs derived using quantile regression and adjusted for age and education.^26^ When determining impairment using cut-offs from the original test description, based on and validated in UK samples,^20^ rates of cognitive impairment were comparable to previous research, (~35% of MND patients).^32^ While the US normative data still require validation against a gold standard full neuropsychological battery, these observations highlight the importance of using appropriate population norms and statistical methods to determine impairment. Furthermore, those who were able to undertake three or more visits had slower rates of disease progression and a lower frequency of cognitive impairment as compared with those with one or two visits, which may have contributed to the lower rates of ALSci in our sample. It is important to note that almost half of those with three or fewer visits were not followed up due to the study ending, rather than drop-out due to cognitive impairment. Given previous reports that cognition is a predictor of survival,^4^ inclusion of individuals with faster disease progression might have identified more individuals with low and declining cognition, particularly as there is an association between cognition and disease stage. However, the problems of attrition in those with faster progression makes longitudinal research problematic. The findings nevertheless show that decline can be detected in a small number of this slower progressing group of patients.

Our observations that for those with cognitive impairment at initial assessment, 83% were impaired on the ALS-specific domains, and that longitudinal decline (among those without a *C9ORF72* repeat expansion) is most marked in the ALS non-specific domains, raise questions about when ALS specific cognitive impairment develops. It is possible that this decline occurred between motor symptom onset and initial assessment (ie, early symptomatic disease) but might also have emerged presymptomatically before emergence of motor deficits (eg, representing a cognitive prodromal stage of disease), highlighting the importance of studying the pre-symptomatic and early symptomatic stages of disease.

Evidence for impairment in cognition in the early stages of symptomatic disease has previously been reported using the King’s Clinical Staging System which stages disease based on increasing involvement of topographic regions.^33^ In the earliest stages of disease (stage 1), 44% of patients had neuropsychological impairment (cognitive and/or behavioural impairment), with 21% showing a specific cognitive impairment on ECAS total scores.^8^ We might speculate that the decline in ALS specific functions may occur, prior to the onset of motor symptoms, in a cognitive prodromal stage of the disease. Studies of asymptomatic *C9ORF72* expansion carriers have suggested impairments in letter fluency,^34 35^ attention and working memory^35^ and executive functioning.^36^ Alternatively, some individuals may have low premorbid cognitive functioning, perhaps indicating an underlying neurodevelopmental disorder or learning disability as suggested by some case studies of individuals with *C9ORF72* who later develop FTD.^37^ Most studies of neuropsychological symptoms in those at genetic risk of ALS have been limited by small sample sizes, cross-sectional designs and the lack of reliable methods of estimating the timing of symptomatic disease onset.

Our findings suggest that there is an association between cognitive decline and *C9ORF72* status. Although the proportion of *C9ORF72* expansion carriers in our sample was small (18%), they tended to have lower scores at first cognitive assessment with greater subsequent decline over time for all three ECAS measures compared with non-carriers. However, *C9ORF72* status did not account for all of those in the low and declining group. In contrast to previous studies,^17 18^ cognitive changes did not differ based on the presence of specific genetic modifiers (*TMEM106B* and *UNC13A*). However prior studies did not use quantitative measures of cognitive functioning and included individuals with more advanced disease.

We also found that higher education was associated with small increases in cognitive performance over time, which raises the question of cognitive reserve. Although some have suggested that certain factors (eg, high education, premorbid IQ, socioeconomic status) are protective against neuropathological damage and cognitive decline,^38^ it is likely that individuals with higher education are able to develop compensatory techniques to counteract progressive impairment. This may result in the maintenance of cognitive functions.

Consistent with previous research,^2^ apathy was the most common behavioural change. It is important to note that approximately a third of participants did not have an informant available which may have lowered the observed rate of ALSbi. Few behavioural changes were recorded in our sample and their presence fluctuated between study visits, making it difficult to explore changes over time. This highlights the difficulties in measuring behaviour change over repeated assessment using available instruments. The ECAS behavioural interview explores whether the behaviours represent a change from premorbid functioning, rather than a previous visit. Therefore, we captured the emergence of new behaviours, but not changes in severity of the behaviour since first assessment. Although every effort was made to ensure that we captured relevant behaviour changes, it is possible that some behaviour symptoms reflect the presence of confounding factors (eg, depression, socioemotional) which were not captured by our behavioural measure. Furthermore, although only one participant reported a history of depression, no data are available on the presence of depressive symptoms which may have contributed to the presence of behavioural symptoms. Future development of the ECAS behaviour screen is needed to ensure a standardised approach to collecting all relevant information.

This study benefits from the use of alternate forms of the ECAS to minimise practice effects. Our analysis focused on the ALS specific, ALS non-specific and ECAS total scores. Although it would be of interest to explore how specific cognitive domains change over time, the ECAS has been designed to focus on ALS specific, ALS non-specific and ECAS total scores as these are used clinically. Due to the brief nature of the ECAS, the range of possible scores within some domains is limited which would make it difficult to detect change in a meaningful way. Assessing cognition using comprehensive neuropsychological assessment in future research would enable the characterisation of cognitive changes at a domain level, although such an approach would likely be beset by attrition given the burden on participants.

## Conclusions

Our findings show that there is heterogeneity in cognitive changes over time. Some people with MND present with deficits in language and executive functioning and although cognition remains stable for most, a small number experience decline across all cognitive domains, especially in memory and visuospatial functioning. This group is associated with fewer years of education and is not entirely explained by *C9ORF72,* although those with this mutation did decline significantly. This suggests that, if present, cognitive functions typically affected in ALS (executive functioning, language, letter fluency) may decline earlier in the disease course and that factors other than *C9ORF72* contribute to these changes. It remains to be determined whether this occurs prior to motor symptoms onset,^39^ which will help identify those who may benefit from early intervention.^40^

## Data Availability

Data are available upon reasonable request. Data that supports the findings of this study are available upon reasonable request from the corresponding author (MB).
