# Supplementary material for: Temporal course of cognitive and behavioural changes in motor neuron diseases
Source: J Neurol Neurosurg Psychiatry. 2023 Oct 12;95(4):316–24. doi: 10.1136/jnnp-2023-331697 (PMC10958376; doi:10.1136/jnnp-2023-331697)
Supplement: Supplementary data [file jnnp-2023-331697supp001.pdf]

eTable 1 Baseline characteristics of individuals with ECAS data at ≤2 versus ≥3 visits.

	**≤2 time points (n=186)**	**≥3 time points (n=237)**	***p* value**
Age	61.3±11.9	59.1±11.2	0.058
Sex (Male)	108 (58%)	143 (60%)	0.709
Years of education	15.7±3.3	15.6±3.1	0.875
Symptom onset to baseline (months)	24.9 [15., 44.0]	31.5 [15.8, 63.7]	0.078
Bulbar symptoms at onset	39 (21%)	56 (24%)	0.594
C9ORF72 [b]	18 (10%)	18 (8%)	0.501
ALSFRS-R	**35 [30, 39]**	**37 [32, 41]**	**0.002**
ΔFRS	**0.49 [0.24, 0.89]**	**0.33 [0.17, 0.59]**	**<0.001**
ALS-FTSD Classification [c]			**<0.001**
ALSci	**17 (9%)**	**15 (6%)**	
ALSbi	**15 (8%)**	**23 (10%)**	
ALScbi	**16 (9%)**	**3 (1%)**	
Baseline cognitive impairment (ALSci/ALScbi)	**33 (18%)**	**18 (8%)**	**0.002**
ECAS Cognition: ALS specific	**80.4±12.5**	**83.6±8.7**	**0.003**
ECAS Cognition ALS non-specific	**27.4±4.3**	**28.4±3.5**	**0.014**
ECAS Cognition: Total scores	**107.8±15.6**	**112.0±10.5**	**0.002**
ECAS Behaviour: Number of affected domains	0.0 [0.0, 1.0]	0.0 [0.0, 1.0]	0.427

Values are mean ± standard deviation, median [25th percentile, 75th percentile]; or n(%).
Significant differences are highlighted in **bold**.
[a] Comparisons are made using t-test, Mann-Whitney U test (continuous) or chi-square test (categorical). Where cells counts are <5, Yates continuity correction has been applied to chi-square test.
[b] *C9ORF72* positive status. Not available for five participants with data at ≤2 time points.
[c] Classified based on available ECAS data for each participant. Participants were not classified as ALS-FTD based on ECAS scores as this requires clinical observations/judgements of change over time.[1] Cognitive impairment determined based on North American cut-offs derived using quantile regression. ALS-FTSD classification unavailable for 4 participants (≤2 time points: n=3; ≥3 time points: n=1) due to missing data for some cognitive tasks.

eTable 2 Frequency of cognitive and behavioural impairment grouped by number of visits with ECAS data

	≥1 visit	≥2 visits	≥3 visits
	**(n=423)**	**(n=327)**	**(n=237)**
**ALS-FTSD Classification by North American Quantile Regression Cut-offs [a]**			
ALSci	47 (11%)	35 (11%)	26 (11%)
ALSbi	71 (17%)	64 (20%)	51 (22%)
ALScbi	29 (7%)	23 (7%)	11 (5%)
ALSci and ALSbi [b]	5 (1%)	5 (2%)	4 (2%)
ALS-FTD	5 (1%)	5 (2%)	4 (2%)
**ALS-FTSD Classification by UK 2 Standard Deviation Cut-offs [a]**			
ALSci	114 (27%)	88 (27%)	67 (28%)
ALSbi	48 (11%)	43 (13%)	33 (14%)
ALScbi	52 (12%)	44 (13%)	29 (12%)
ALSci and ALSbi [b]	5 (1%)	5 (2%)	4 (2%)
ALS-FTD	5 (1%)	5 (2%)	4 (2%)
**ECAS – Behaviour [c]**	**(n=345)**	**(n=243)**	**(n=165)**
Disinhibition	25 (7%)	21(9%)	18 (11%)
Apathy	95 (27%)	73 (30%)	51 (31%)
Loss of sympathy/empathy	67 (19%)	56 (23%)	39 (24%)
Perseveration	46 (13%)	40 (16%)	27 (16%)
Hyperorality	37 (11%)	31 (13%)	23 (14%)

Values are n (%).
Based on evidence of impairment for at least one visit.
[a] Classified based on available ECAS data for each participant. PLS and PMA participants were also classified using the same criteria based on prior evidence of comparable neuropsychological profiles. Diagnosis of ALS-FTD is based on clinical examination.
[b] Classified as ALSci and ALSbi at different visits.
c N=78 did not have ECAS behaviour data available at any visit. Participants may have multiple behaviour symptoms therefore proportions will not add up to 100%.

eTable 3 Cognitive change over time: Factors associated with changes in ECAS scores.

	β (SE)	95% CI
**ALS Specific Scores**		
Intercept	87.15 (1.17)	84.85, 89.45
Time [a]	-0.02 (0.03)	-0.08, 0.05
Education [b]	2.09 (0.72)	0.68, 3.51
C9ORF72[c]	-1.63 (2.18)	-5.91, 2.66
Time [a] x *C9ORF72* [c]	-0.32 (0.16)	-0.62, -0.01
Time [a] x Education [b]	0.10 (0.04)	0.02, 0.18
**ALS Non-specific Scores**		
Intercept	28.63 (0.44)	27.78, 29.49
Time [a]	0.05 (0.02)	0.02, 0.08
C9ORF72 [c]	0.34 (0.84)	-1.30, 1.98
Time [a] x *C9ORF72* [c]	-0.17 (0.07)	-0.32, -0.03
**ECAS Total Scores**		
Intercept	115.69 (1.43)	112.88, 118.50
Time [a]	0.04 (0.04)	-0.04, 0.12
C9ORF72 [c]	-1.09 (2.63)	-6.24, 4.06
Time [a] x *C9ORF72* [c]	-0.50 (0.20)	-0.89, -0.12

All models are adjusted for baseline ∆FRS-R, baseline age, sex, education, bulbar symptoms at onset and *C9ORF72* status.
β (SE) = Regression coefficient (standard error) from mixed model analysis.
[a] Follow-up duration, in months
[b] In 4-year increments, and mean centred
[c] *C9ORF72* positive status

eTable 4 *TMEM106B* and *UNC13A* SNP frequencies

SNP ID	Genotypes	*C9ORF72* positive (n=18)	*C9ORF72* negative (n=219)
rs3173615	GG	5 (28%)	46 (21%)
	CG	9 (50%)	103 (47%)
	CC	4 (22%)	69 (32%)
rs12608932	CC	2 (11%)	22 (10%)
	AC	9 (50%)	95 (44%)
	AA	7 (39%)	100 (46%)

eTable 5 Cognitive profiles of latent class subgroups

	Subgroup-1 (High-baseline-upward)		Subgroup-2 (Intermediate-baseline-stable)		Subgroup-3 (Low-baseline-downward)	
	**Estimate (SE) [a]**	***p* value**	**Estimate (SE) [a]**	***p* value**	**Estimate (SE) [a]**	***p* value**
**ALS Specific scores**						
Intercept	90 (0.48)	<0.001	80 (0.60)	<0.001	67 (1.09)	<0.001
Time [b]	0.08 (0.04)	0.040	-0.10 (0.05)	0.033	-0.21 (0.13)	0.116
**ALS Non-specific scores**						
Intercept	30 (0.25)	<0.001	27 (0.35)	<0.001	22 (0.59)	<0.001
Time [b]	0.08 (0.02)	<0.001	0.02 (0.03)	0.535	-0.22 (0.06)	<0.001
**ECAS Total scores**						
Intercept	119 (0.56)	<0.001	108 (0.78)	<0.001	91 (1.34)	<0.001
Time [b]	0.17 (0.05)	<0.001	-0.11 (0.06)	0.084	-0.58 (0.16)	<0.001

[a] Regression coefficient (standard error) from latent class growth analysis
[b] Points per month

eTable 6 Predictors of cognitive profiles in latent class subgroups

	Intermediate baseline-stable subgroup OR (95%CI)	Low baseline-downward subgroup OR (95%CI)
**ALS specific**		
Age (years)	**1.05 (1.01 – 1.09)**	1.03 (0.97 – 1.08)
Sex, male	**2.26 (1.09 – 4.70)**	2.78 (0.80 - 9.67)
Education (years) [a]	**0.46 (0.28 – 0.74)**	**0.31 (0.14 – 0.73)**
Bulbar symptoms at onset	1.10 (0.47 – 2.55)	1.52 (0.42 – 5.58)
C9ORF72 expansion carrier	2.65 (0.36 – 19.49)	4.95 (0.63 – 38.83)
ΔFRS	2.05 (0.75 – 5.64)	3.36 (0.90 – 12.55)
**ALS non-specific**		
Age (years)	**1.15 (1.03 – 1.29)**	1.05 (0.99– 1.11)
Sex, male	2.81 (0.72 – 10.96)	0.34 (0.10 – 1.17)
Education (years) [a]	0.55 (0.27 – 1.09)	0.47 (0.21 – 1.05)
Bulbar symptoms at onset	2.06 (0.56 – 7.54)	2.22 (0.66 – 7.49)
C9ORF72 expansion carrier	3.74 (0.36 – 38.32)	1.25 (0.18 – 8.70)
ΔFRS	1.14 (0.23 – 5.59)	2.33 (0.63 – 8.64)
**ECAS total**		
Age (years)	**1.07 (1.03 – 1.11)**	1.03 (0.97 – 1.09)
Sex, male	**2.29 (1.05 – 4.98)**	1.13 (0.34 – 3.77)
Education (years) [a]	**0.37 (0.21 – 0.64)**	**0.39 (0.16 – 0.93)**
Bulbar symptoms at onset	1.73 (0.71 – 4.23)	1.69 (0.46 – 6.14)
C9ORF72 expansion carrier	1.06 (0.18 – 6.25)	2.88 (0.54 – 15.34)
ΔFRS	2.05 (0.73 – 5.73)	2.89 (0.78 - 10.65)

Reference = Subgroup-1 (High baseline -upward)
Results highlighted in **bold** are significant at $p<0.05$.
[a] In 4-year increments
